# Licorice consumption as a cause of posterior reversible encephalopathy syndrome: a case report

**DOI:** 10.1186/cc10040

**Published:** 2011-02-18

**Authors:** Eduard J van Beers, Jan Stam, Walter M van den Bergh

**Affiliations:** 1 Department of Internal Medicine, Academic Medical Center, University of Amsterdam, Meibergdreef 19, Amsterdam NL-1100 DD, the Netherlands; 2 Department of Neurology, Academic Medical Center, University of Amsterdam, Meibergdreef 19, Amsterdam NL-1100 DD, the Netherlands; 3 Department of Intensive Care, Academic Medical Center, University of Amsterdam, Meibergdreef 19, Amsterdam NL-1100 DD, the Netherlands

## Abstract

**Introduction:**

A 49-year-old woman was admitted to our hospital because of thunderclap headache and blurred vision. At the time of presentation, her blood pressure was 219/100 mmHg, her arterial pH was 7.64 and her potassium level was 2.7 mM/l.

**Methods:**

The combination of sequential computed tomography (CT) and the triad of hypertension, hypokalemia and metabolic alkalosis in this patient suggested the diagnosis. Supplementary anamnesis and long-term follow-up confirmed it.

**Results:**

Brain computed tomography imaging showed minor bleeding in the left Sylvian fissure and bilateral occipital edema, suggestive of posterior reversible encephalopathy syndrome (PRES). Repeated brain CT after 10 days showed a complete resolution of radiological signs. The patient informed us that she had quit smoking 2 weeks ago and had started consuming large amounts of licorice instead of smoking. After she abandoned licorice consumption, her blood pressure normalized. Her latest blood pressure reading was 106/60 mmHg without the use of any antihypertensive drugs.

**Conclusions:**

To the best of our knowledge, this is the first case report describing licorice consumption as a cause of PRES. Glycyrrhizic acid, a component of licorice, inhibits 11β-hydroxysteroid dehydrogenase and subsequently causes mineralocorticoid excess. Mineralocorticoid excess in turn causes high blood pressure and ultimately gives rise to malignant hypertension. Physicians should remember that licorice use is a very easy-to-treat cause of hypertension, hypertensive encephalopathy and PRES.

## Introduction

The reversible posterior leukoencephalopathy syndrome, or posterior reversible encephalopathy syndrome (PRES), was first described by Hinchey *et al*. in 1996 [1]. It is characterized by symptoms of acute neurological (often visual) symptoms combined with brain imaging findings correlating with vasogenic edema and clinical or radiologic proof of reversibility [2,3].

Since the first report in 1996, numerous patients with this syndrome have been described [2,4-6]. As the syndrome can occur in children as well as in adults, the mean age in all large case series is typically approximately 45 years, with a range involving all ages. Seizures are a presenting symptom in 67% to 87% of cases. Other frequently occurring symptoms include combinations of headache (26% to 53%), visual impairment (20% to 39%) or altered mental status (28% to 92%). Also, in children, seizures (85%), headache (46%) and visual disturbances (52%) or altered mental status (60%) are frequent presenting symptoms [7].

Common associated conditions in patients with PRES are hypertension, preeclampsia-eclampsia, acute or chronic renal failure, infection, sepsis or multiple organ dysfunction, autoimmune disease and treatment with chemotherapeutic or immunosuppressant agents (the most reported drugs are tacrolimus and cyclosporine) [2]. Often more than one condition is involved in a certain case. For instance, a patient with Wegener granulomatosis who is taking immunosuppressive medication and presents with a renal crisis provoked by infection has four conditions associated with PRES.

Computed tomography (CT) or magnetic resonance imaging of the brain typically demonstrates focal regions of symmetric hemispheric edema. Although the occipital lobe is predominantly involved, hence its name, lesions can also involve or be restricted to the parietal or frontal lobe.

The clinical hallmark of the syndrome is an acute rise in blood pressure, which is more important than the blood pressure level itself, although in the vast majority (70% to 86%) of cases, acute hypertension is exists at the time of presentation. Clinical symptoms resolve after prompt treatment of hypertension. Edema usually reverses within days to weeks [2].

## Materials and methods

### Patient details

A 49-year-old woman with a history of migraine presented to the emergency room with a thunderclap headache, initially without other symptoms but later followed by visual impairment. She remained conscious and had no complaints besides headache. She uses pantoprazole and had quit smoking 2 weeks earlier. She had been well until the development of the headache, and she had not experienced any trauma.

**Figure 1:**
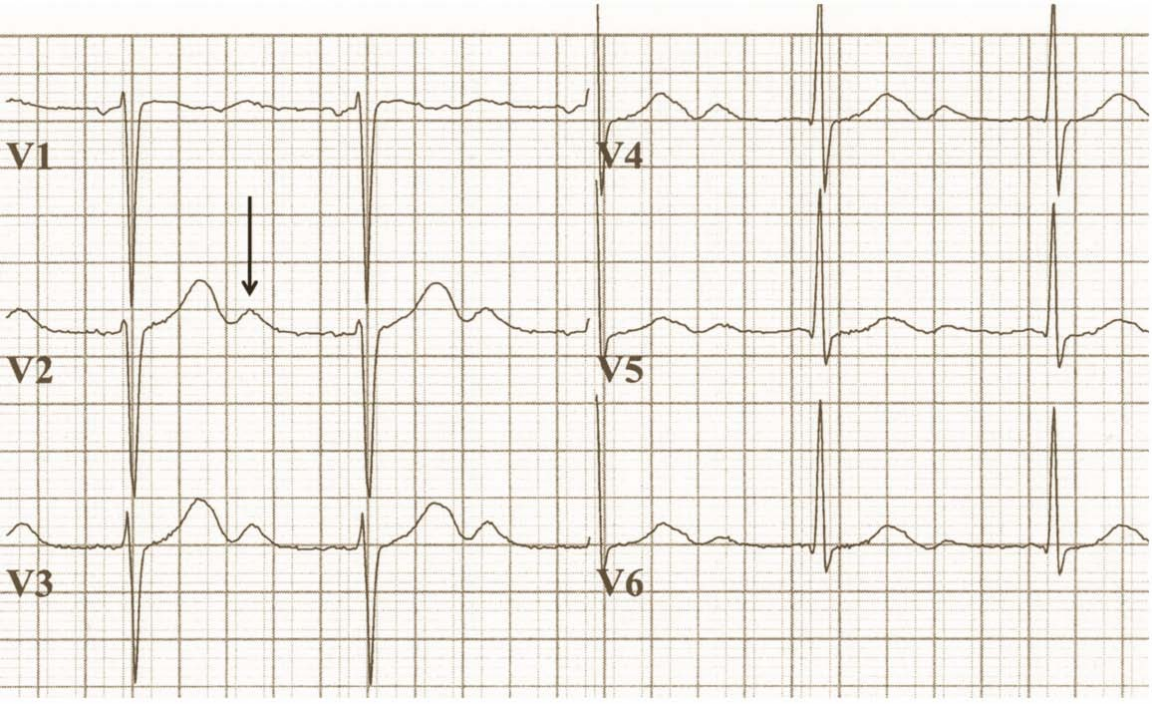
**The patient's electrocardiogram shows a prominent U-wave (arrow) at a potassium concentration of 2.7 mM/l**. The precordial leads matched the voltage criteria for left ventricular hypertrophy [9].

Her physical examination revealed that she had a pulse of 62 beats/minute and blood pressure of 219/100 mmHg. Her motor and sensory responses were normal. Cardiac and pulmonary sounds were normal. The ocular fundus appeared normal on the basis of ophthalmoscopy. Her electrocardiogram showed decreased T wave amplitude, ST segment depression and the presence of a marked U wave (Figure 1), which were suggestive of severe hypokalemia [8]. The precordial leads matched the voltage criteria for left ventricular hypertrophy [9]. Laboratory analysis showed a low serum level of potassium at 2.7 mM/l. Her sodium and magnesium levels were normal. Arterial blood gas analysis showed a mixed metabolic and respiratory alkalosis, with an arterial pH of 7.64 and a bicarbonate level of 27.4 mM/l. However, within hours after presentation, the respiratory component resolved, leaving only metabolic alkalosis.

### Ethics

The patient gave her written permission for us to publish this manuscript.

## Results

### Evaluation

**Figure 2:**
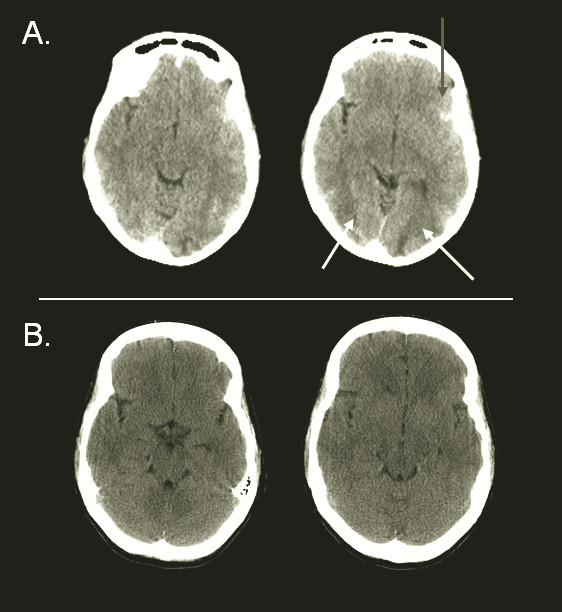
**(A) Brain computed tomography (CT) performed at the time of presentation showed a small amount of blood in the left Sylvian fissure (gray arrow) and bilateral edema in the occipital regions (white arrows), suggestive of posterior reversible encephalopathy syndrome**. **(B) **Brain CT performed 10 days later shows a total resolution of radiological signs.

A brain CT scan showed a small hemorrhage in the left Sylvian fissure and bilateral edema in the occipital regions (Figure 2). Because of the acute onset of the headache and the finding of subarachnoid blood, CT angiography was performed, but no aneurysm was found.

### Treatment

The diagnosis of PRES was made, and the patient was admitted to the intensive care unit (ICU) for monitoring of blood pressure and neurological status as well as treatment of hypertension with labetolol. The patient quickly recovered and was discharged from the hospital after a few days. Ten days after her initial presentation, repeated brain CT showed complete resolution of the radiological signs (Figure 2).

### Licorice consumption

The triad of hypertension, hypokalemia and metabolic alkalosis raised the suspicion of mineralocorticoid excess. Upon admission at the ICU, the patient told us that she had stopped smoking 2 weeks previously and that since then she had started consuming large amounts of licorice as a substitute for her smoking addiction. Glycyrrhizic acid, a component of licorice, inhibits 11β-hydroxysteroid dehydrogenase and subsequently causes mineralocorticoid excess.

### Follow-up

The patient was seen in the outpatient clinic several times after the incident. After she had successfully stopped smoking, she also managed to abandon her licorice addiction. Her latest blood pressure reading was 106/60 mmHg without the use of any antihypertensive drugs. She was normokalemic, and her metabolic alkalosis had normalized.

## Discussion

The cause of PRES is not yet understood. Hypertension with failed cerebral autoregulation and hyperperfusion remains a popular consideration for the development of brain edema. PRES is also seen in the absence of hypertension, and in many cases the degree of hypertension does not typically reach the limit of cerebral autoregulation. This seems to contradict the defective autoregulation hypothesis. However, preexisting dysfunction of the vascular endothelium, as reported in association with immunosuppressive therapy or in preeclampsia and eclampsia, may predispose patients to failure of cerebral autoregulation and encephalopathy, even after relatively small increases in blood pressure.

The triad of hypertension, hypokalemia and metabolic alkalosis can be caused by mineralocorticoid excess. The reason for this presentation in our patient was her excessive consumption of licorice to control her craving for cigarettes. Compared to that in other countries, licorice consumption in the Netherlands is very high: on average 2 kg per person annually. Also, licorice tea is becoming more popular [10]. Ingestion of licorice is a well-known, though relatively uncommon, cause of hypertension due to hypermineralocorticoidism. Although its exact incidence is not known, some studies have shown that licorice-induced hypertension may be the cause of 3% of all hospital cases involving symptomatic hypertension [11].

Glycyrrhizic acid, a component of licorice, produces hypermineralocorticoidism through the inhibition of 11β-hydroxysteroid dehydrogenase. The presence of partial 11β-hydroxysteroid dehydrogenase deficiency could explain why some people are susceptible to even low doses of glycyrrhizic acid. However, many other conditions could also cause acquired inhibition of the enzyme, such as chronic renal failure, hypothyroidism and some kinds of essential hypertension [12].

Patients with mineralocorticoid excess have impaired endothelium-dependent vascular reactivity, which is correlated with the level of mineralocorticoids and not with the level of hypertension [13]. As discussed above, impaired cerebral endothelium-dependent vascular reactivity could contribute to the development of PRES in the setting of high blood pressure in these patients. Thus, hypermineralocorticoidism due to licorice consumption could hypothetically contribute to the development of PRES in two pathophysiologically distinct ways: first and foremost by induction of hypertension, but possibly also by impairing cerebral endothelial function.

## Conclusions

The association of licorice and hypertensive encephalopathy has been described before, and hypertensive encephalopathy can be seen as part of the spectrum of PRES symptoms [14-16]. Physicians should remember that licorice use may be the underlying cause of hypertension, hypertensive encephalopathy and PRES. Moreover, licorice addiction is probably easier to treat than a smoking habit.

## Key messages

• Licorice consumption is a potential cause of PRES.

## Abbreviations

CT: computed tomography; PRES: posterior reversible encephalopathy syndrome.

## Competing interests

The authors declare that they have no competing interests.

## Authors' contributions

All authors participated in the design of the study, collection of the data, analysis of the data and critical review of the paper.
